# Severe Acute Respiratory Syndrome-Coronavirus-2 (SARS-CoV-2): A Perspective Through the Lens of the Veterinary Diagnostic Laboratory

**DOI:** 10.3389/fvets.2020.576267

**Published:** 2020-09-25

**Authors:** Tracy Stokol, Denise McAloose, Karen A. Terio, Francisco J. Salguero

**Affiliations:** ^1^Department of Population Medicine and Diagnostic Sciences, College of Veterinary Medicine, Cornell University, Ithaca, NY, United States; ^2^Wildlife Conservation Society, Zoological Health Program, Bronx Zoo, Bronx, NY, United States; ^3^Zoological Pathology Program, Department of Veterinary Clinical Medicine, College of Veterinary Medicine, University of Illinois, Brookfield, IL, United States; ^4^Public Health England, Porton Down, Salisbury, United Kingdom

**Keywords:** COVID-19, diagnostic testing, serology, molecular genetics, viral infection, wildlife, zoological animals, public safety

## Abstract

The SARS-CoV-2 pandemic has resulted in unprecedented challenges to veterinary diagnostic laboratories. These challenges include partial or complete shutdowns, interrupted courier services, disruptions in workflow and diagnostic testing, new physical distancing practices, protocol development or enhancement for handling samples from high-risk or susceptible species, and fulfilling requirements for pre-test permission approval from state and federal veterinary agencies, all of which have been implemented to prevent or minimize exposure and transmission of SARS-CoV-2 locally or regionally. As in people, SARS-CoV-2 infects animals through direct animal-to-animal contact and aerosol transmission between animals. Humans can also infect pets or other animals in their care and, although human-to-human transmission is the main route of viral spread in people, infected animals and specimens of their bodily fluids or tissues are a potential source of infection for veterinarians and technical or laboratory personnel that are handling them. In this perspective, we discuss how SARS-CoV-2 has necessitated rapid changes in laboratory operation to minimize zoonotic risk to personnel and to implement tests for identifying the virus in animals. The pandemic has highlighted the adaptability and quick response of veterinary diagnosticians to an emerging infectious disease and their critical role in maintaining animal health, while synergizing with and protecting human public health.

## Introduction

A once in a lifetime global pandemic due to SARS-CoV-2 is upon us and veterinarians are rising to the challenge, responding quickly to this novel zoonotic disease. Veterinarians are trained in comparative medicine across species and to always consider infectious diseases when examining or treating animals or handling bodily fluids and tissues for diagnostic testing. As such, veterinarians have the expertise to contribute to discussions and research related to disease pathogenesis as well as to concerns of disease transmission from animals-to-humans and humans-to-animals, with the attending health implications for animals. For veterinary pathologists and diagnosticians, the outbreak has necessitated rapid implementation of molecular and serologic assays as screening, diagnostic and research tools for SARS-CoV-2, enhancement of protocols to ensure safety of laboratory personnel handling fluids and tissue from susceptible, suspect, or infected animals, and reconfiguration of laboratory spaces with modification of procedures to facilitate operation while maintaining local, regional, and national guidelines for personal protection, including physical distancing.

Veterinary diagnostic laboratories are adept at analyzing many different specimens from a wide array of species using standard operating procedures, akin to those in human medical laboratories. These procedures include mandatory personal protective equipment (PPE), such as gloves and dedicated clothing, and engineering controls that vary depending on the biosafety level (BSL) concern and risk assessment. Laboratories are also well-equipped to handle samples that may have come from an animal infected with an organism of high zoonotic potential, such as cerebrospinal fluid (e.g., rabies), blood and tissue (e.g., anthrax), and urine (e.g., leptospirosis). SARS-CoV-2 is a new addition to this existing list of zoonotic diseases that pose a risk to laboratory personnel, although cases of laboratory-acquired SARS-CoV-1 infections are rare in human medicine (1, 2) with none-to-date reported for SARS-CoV-2 in human or veterinary diagnostic laboratories. Nevertheless, given the frequently unknown infectious status of animals or their owners, veterinary diagnostic laboratories have re-evaluated protocols to further reduce risk to personnel handling samples, particularly from species susceptible to SARS-CoV-2.

## Susceptible Animal Species

Domestic and non-domestic felids, dogs, ferrets, mink, non-human primates, and hamsters can be naturally or experimentally infected with the virus, with shedding of variable degree and duration and evidence of inter-individual transmission (3–19). Subsequent to the original animal-to-human transmission event and resulting human-to-human transmission, the current infection paradigm is that companion and non-domestic farmed or captive animals acquire the virus from humans. However, the infection rate in pet animals appears low. In a study from Italy, viral RNA was not detected in nasopharyngeal, nasal and/or rectal swabs from 839 pet dogs and cats, including 76 animals with clinical signs of respiratory disease. Of the tested animals, 14% were from households with COVID-19 (20). Serum neutralizing antibodies was detected in 3–4% of animals, although a higher proportion of serologically-positive dogs were from COVID-19 vs. non-COVID-19 households. In two other studies of 21 (dogs and cats) and 23 (dogs, cats, rabbits, and a guinea pig) pet animals from France (21) and Spain (22), viral RNA was detected in a nasopharyngeal swab from one cat in the Spanish study (22). In contrast, 15% of 143 pet and stray domestic cats had serum antibodies to SARS-CoV-2 in a study conducted in Wuhan, China, after the outbreak; cats with the highest titers were from households with COVID-19 (23). It is unclear if these differences relate to actual exposure or variability in performance of the applied tests. To date, there has only been one report of suspected animal-to-human transmission from farmed mink (24), suggesting that zoonotic transmission to people is still possible when working with high numbers of susceptible animals in close confined quarters. Based on gene sequencing, the ancestral SARS-CoV-2 virus is thought to have its origin in a species of bat with subsequent evolution to its current form in one or more intermediate animal hosts, possibly including pangolins (25, 26). The spike protein on the virus envelope facilitates cell entry by binding to the transmembrane protein, angiotensin-converting enzyme-2 (ACE2). Amino acid sequencing of the spike protein binding domain and ACE2 and *in silico* modeling of the spike protein binding to ACE2, is being used to explore susceptibility of experimental and domestic animals to SARS-CoV-2 and identify potential wildlife hosts (6, 27–30). Two modeling studies predict that equine, camelid, bovine and ovine ACE2 will bind SARS-CoV-2 (27, 30), however no infectivity studies have been reported as yet for these species. Similarly, *in vitro* studies of the spike protein (in pseudotyped virions) binding to cloned ACE2 and infection studies of cell lines derived from different species suggest that rabbits may be infected with the virus (31, 32). However, as shown for pigs (12, 33), modeling and *in vitro* studies do not always translate into susceptibility *in vivo*.

## Laboratory Handling of Specimens That May Contain Sars-CoV-2

Specimens with the highest biohazardous risk to laboratory personnel are respiratory secretions and tissues that contain infectious virus (5–10, 12, 16, 19, 34–37). Direct mucosal contact with or inhalation of respiratory droplets or aerosols are the primary routes of infection (9, 19, 38), with intranasal administration being the experimentally used counterpart of natural infection (6, 7, 9, 10, 12, 19, 34, 37, 39). A brief low-level viremia has been documented in several human patients (40–43) and experimentally infected ferrets (9), macaques (10), and hamsters (6). In humans, viral RNA can be found in feces and rarely in urine (36, 41, 43, 44), with one report of infectious virus in feces (45). Viral RNA has been amplified from feces in domestic and wild felidae (12, 16), feces and urine from ferrets (9) and feces from monkeys (46), with infectious virus being isolated from feces from non-domestic cats (16), ferrets (9), and monkeys (46). While feces, urine and blood may not contain as much intact virus as respiratory samples, they are still considered infectious, but likely pose a low risk to laboratory personnel. Longitudinal studies of the infectious nature of respiratory secretions, other bodily fluids, such as saliva, and feces or rectal/anal tissue are needed across a range of domestic and non-domestic animals and livestock to develop a clear understanding of the relative risk and the duration of risk that these biomaterials pose to owners, laboratory personnel, and contact animals.

When handling samples from SARS-CoV-2 susceptible animals for routine laboratory testing, guidelines established by government entities are followed [e.g., Centers for Disease Control and Prevention (47) and Public Health England Gov. UK. (48)]. These guidelines recommend standard protocols, with additional precautions for higher risk procedures that can generate aerosols, such as centrifugation or autopsies. Veterinary laboratories typically operate under BSL-2 conditions (49), with most samples for microbiological or molecular diagnostic procedures being handled within a microbiological or biological safety cabinet (BSC). Inactivation steps are often included in protocols, but are not always feasible or desirable. For cytologic samples, optimal preparation of tracheal wash and bronchoalveolar lavage fluids often requires centrifugation-based concentration. Additional protective measures for handling such specimens could include face protection for benchwork, using BSC for preparing slides, only preparing direct smears for examination (feasible for tracheal washes but not low cellularity bronchoalveolar lavages), using centrifuges with sealed rotors (cytocentrifuges), operation of or opening centrifuges within BSC, and alcohol- or heat-fixing slides (which may adversely affect smear quality). Smears of respiratory secretions on unstained unfixed glass slides may contain live virus for several hours to perhaps days (50, 51), however, virus is unlikely to be aerosolized from the slides and unstained/unfixed slides pose low risk if handled with appropriate PPE. It is assumed slide staining, particularly with alcoholic-based stains, will inactivate virus, but this theory remains to be tested. Recommendations for autopsies on suspect COVID-19 human patients include N95 masks, eye protection, conducting minimally invasive autopsies (e.g., ultrasound-guided needle biopsies), and delaying autopsies until confirmatory testing is complete (52). Similar recommendations have not been published for post-mortem examination of animals, however existing protocols for highly pathogenic zoonotic diseases can be modified to include targeted sample collection for SARS-CoV-2 in suspect cases with approval (53, 54) and guidance on collection (47, 48, 55, 56).

## Testing for SARS-CoV-2 in Laboratory Samples for Animals

Veterinary diagnostic laboratories play an essential role in disease outbreaks through testing for the organism and antiviral immunity and through educating clients about the relevance of positive and negative results. Testing is the core of epidemiologic studies of prevalence and spread, associating infection with disease, and identifying susceptible hosts or vectors. Local, state, national, commercial, academic, and research veterinary laboratories quickly adapted existing methods and validated tests to identify SARS-CoV-2 RNA or antigen and serologic responses in animals. In the early pandemic phase, several veterinary laboratories were approved for SARS-CoV-2 testing on human samples in the event that expanded capacity was needed by the human health community (57, 58). Currently, 9 of the 59 veterinary diagnostic laboratories within the National Animal Health Laboratory Network (NAHLN) have been certified by the Department of Health and Human Services to perform testing of human samples in the United States (59), demonstrating how readily veterinary laboratories can be repurposed at times of need. Many laboratories donated equipment, reagents, and supplies as part of their pandemic efforts.

The current standard for detecting active SARS-CoV-2 infection and determining infection prevalence is through real time reverse transcriptase-polymerase chain reaction (rRT-PCR) for SARS-CoV-2 RNA. The test is exquisitely sensitive and specific. However, care must be taken to correctly interpret results and avoid false positive or negative results. False positive results can occur through cross-sample contamination during collection or testing (60–62). Confirmatory testing, ideally from a different laboratory, would increase confidence in positive results. A positive rRT-PCR reaction does not necessarily indicate replication-competent virus and an infectious patient, since degraded RNA may be detected (60). Virus isolation can verify that RNA equates to infectious virus in natural and experimental infections, but requires BSL-3 facilities and suitable cell lines for infection (e.g., Vero cells). False negative rRT-PCR results can be due to patient factors (e.g., intermittent shedding), sample collection (e.g., wrong timing, insufficient or inadequate specimens), sample handling (e.g., RNA degradation with storage), or test limitations (e.g., inadequate RNA extraction, insensitive primers, RNA levels below detection limits in early or low-level infections) (60–62). Viral RNA can be detected *in situ* using RNA hybridization and virus-specific probes in research studies (7, 10, 11, 16, 42). As for any laboratory test, it is critically important to include positive and negative controls and verify specificity for SARS-CoV-2 to prevent cross-reaction with other coronaviruses, minimize the likelihood of false positive or negative reactions and verify test performance.

Immunologic-based tests are used to detect SARS-CoV-2 viral antigen or antibodies indicative of a serologic response to infection. Immunohistochemical application of monoclonal or polyclonal antibodies against the spike and nucleocapsid proteins has been invaluable for determining viral tissue tropism in experimental studies (5, 6, 8, 9, 11, 34, 39, 42). However, the presence of viral antigen in bodily fluids does not necessarily indicate infectious virus, because antibodies can bind to inactivated virus, such as in formalin- or alcohol-fixed samples. Serologic assays for anti-SARS-CoV-2 antibodies can be performed with ELISA, multiplex assays or other platforms, including those designed for point-of-care use. However, a positive reaction does not necessarily equate to immunity and, like any diagnostic test, false positive and negative reactions occur, with a higher likelihood of false positive reactions in regions with low prevalence. In veterinary settings, the lack of species-specific secondary antibodies can be a major limitation for serologic testing. Thus, serum neutralization assays are often used to detect antibodies, especially when testing samples from non-domestic wildlife or zoological animals (16). Antibodies for immunologic testing should be thoroughly validated, as described for immunohistochemical staining (63). The sensitivity and specificity of immunologic-based tests depends on antibody avidity, the antigenic epitopes detected by the antibodies, and detection method; different assays may not yield equivalent results. For example, a recent meta-analysis comparing performance of serologic SARS-CoV-2 assays in human patients showed that fluorescence- or point-of-care chromatographic-based assays were less sensitive than ELISA- or chemiluminescence-based assays and ELISAs targeting the spike protein were more sensitive (albeit with overlapping confidence intervals) than those against the nucleocapsid protein (64). Larger scale studies to evaluate assays across platforms are underway in human medicine and are yet to be done for animal testing. Veterinary diagnostic laboratories have a wealth of archived and fresh samples from multiple species to use for test validation and determining repeatability and performance of currently used and newly developed assays. One program to verify performance across veterinary diagnostic laboratories (Inter-Laboratory Comparison Evaluation) is being established by the United States Food and Drug Administration's Veterinary Laboratory Investigation and Response Network on behalf of the NAHLN (Dr. François Elvinger, personal communication). Recognizing the need for the rapid development of SARS-CoV-2 tests, future goals should include independent assessment of test performance to support developers' claims of sensitivity and specificity, testing for inter-laboratory agreement, production of high-quality control materials for internal and external proficiency testing, and open-access publication or reporting of methods and test performance statistics. Such studies and transparency are necessary to inform clients and the public of test performance and increase confidence in test results, whether performed in diagnostic laboratories or at point-of-care.

## Discussion

SARS-CoV-2 is a new addition to the list of zoonotic agents that might be present in animal specimens handled and tested in veterinary diagnostic laboratories. Procedures that were rapidly put in place in an emergent situation to deal with high-risk samples will need to be revisited and refined as more information about the virus comes to light. No doubt the virus will be a hot topic of conversation at future pathology and diagnostic association scientific meetings, where shared experiences, successes, and failures will help inform policy and protocols. Ideally, these discussions will lead to the development and adoption of industry-wide or consensus standards for sample handling and testing, result reporting and interpretation, and archiving and disposal of high-risk specimens. It is also likely that the list of susceptible animal species will continue to grow as additional natural infections are identified and knowledge is acquired from research studies on domestic, wild, and zoological animals. Many questions about the virus remain to be answered, such as how long the virus persists in laboratory samples, how best to inactivate the virus in routine preparations (e.g., smears of potentially infected material on glass slides) while maintaining diagnostic quality, and whether routine staining of such slides inactivates the virus. Veterinary diagnosticians and researchers are well-poised to take advantage of their substantial available resources to perform these studies.

In the United States, routine SARS-CoV-2 testing is currently not recommended in animals (53, 54, 65). Arguments against widespread testing include the lack of understanding of the meaning of positive or negative results, lack of clarity on appropriate preventative or therapeutic interventions for animals with positive results, and concerns for animal and human welfare, such as pet abandonment or euthanasia, killing of wildlife populations, and disruption of the human-animal bond when separating owners from pets. In the early days of the pandemic, there was negative public perception for testing animals when human facilities needed reagents and supplies for testing people. Current animal testing guidelines recommend first ruling out other conditions, justification of need, and approval by state veterinarian or public health officials, with confirmation of presumptive positive results by the National Veterinary Services Laboratory in Iowa (53). The World Organization of Animal Health has defined SARS-CoV-2 as a reportable emerging disease (66). However, the situation is rapidly evolving and there remains a need to better understand the true prevalence of SARS-CoV-2 in companion, working or food animals, and zoological and wildlife populations, which can be accomplished by studies involving broader testing. Improved knowledge of true prevalence would better inform veterinary clinical and laboratory practice and owners of such animals, enabling science-based recommendations for risk assessments. More testing will also allow us to refine our understanding of virus epidemiology and establish whether and which animals are reservoirs of the virus, information critical to break transmission chains and protect animal and human health. SARS-CoV-2 testing in animals is generally performed on nasopharyngeal swabs, fecal samples or rectal swabs (3, 5, 7–10, 12, 16, 19); testing on saliva may be an additional option in animals (9, 35). Fecal sampling is an appealing non-invasive collection method, allowing ready surveillance of at-risk wildlife or zoological animals, albeit at the risk of reduced sensitivity due to less viral RNA (16). Shedding periods may extend beyond the period of clinical signs, thus fecal testing may help us understand how long shedding persists, which will contribute to risk assessments and epidemiological investigations into transmission.

The mission of many veterinary diagnostic laboratories includes the provision of diagnostic testing for disease identification and maintenance of animal health, food security, and human public health. Sustaining efficient ongoing operations is essential to allow veterinary diagnostic laboratories to continue to fulfill this mission. The current pandemic complicates delivery of this mission, however veterinary laboratories have shown remarkable adaptability and innovation when handling this unprecedented crisis. At least in the near term, and potentially until effective vaccine(s) are available, some laboratories may institute regular testing of personnel to minimize the likelihood of localized outbreaks among staff and prevent major disruptions in laboratory services. The pandemic also offers a rich opportunity for veterinary diagnosticians and pathologists to contribute to testing and research, through primary or collaborative efforts.

Veterinary diagnostic laboratories have responded to the call of the SARS-CoV-2 pandemic by rapidly establishing new or enhancing existing protocols to ensure safe laboratory practices and implementing and validating molecular- and immunologic-based assays for virus detection. They have also helped to expand testing capacity for human patients, all while concurrently performing routine diagnostic testing for other animal diseases and often with reduced staffing related to governmental or organizational mandates. The combined expertise of anatomic and clinical pathologists, molecular diagnosticians, and virologists, working collaboratively as teams with highly qualified and dedicated personnel, positions veterinarians to be key partners in understanding natural disease that impacts human health, such as this coronavirus pandemic, as well as for leading or collaborating with discovery efforts into viral pathogenesis, diagnostic testing, and treatment.

## Data Availability Statement

The original contributions presented in the study are included in the article/supplementary material, further inquiries can be directed to the corresponding author.

## Author Contributions

TS wrote the article, which was edited by DM, KT, and FS. All authors contributed to the article and approved the submitted version.

## Conflict of Interest

The authors declare that the research was conducted in the absence of any commercial or financial relationships that could be construed as a potential conflict of interest.
